# Comparative Transcriptome Analysis Identifies Candidate Coordinated Defense Mechanisms in Corn Stalk Rot Resistance

**DOI:** 10.3390/biology15212033

**Published:** 2026-11-08

**Authors:** Peng Huang, Linlin Song, Jie Zhang, Deyi Yu, Zhaohua Zeng, Benjin Li, Jinai Yao

**Affiliations:** Fujian Key Laboratory for Monitoring and Integrated Management of Crop Pests, Institute of Plant Protection, Fujian Engineering Research Center for Green Pest Management, Fujian Academy of Agricultural Sciences, Fuzhou 350013, China

**Keywords:** corn stalk rot, *Fusarium* resistance, defense mechanisms, hub genes

## Abstract

Corn stalk rot (CSR) is a devastating soil-borne disease, but the transcriptional basis of constitutive resistance remains unclear. Here, we performed comparative transcriptome analysis of the CSR-resistant maize cultivar Qi319 (JR) and the CSR-susceptible maize cultivar Ye478 (JS) under pathogen-challenged conditions. A total of 6009 differentially expressed genes (DEGs) (3074 upregulated, 2935 downregulated in JR) were identified, with JR exhibiting a higher proportion of low-expression genes. Functional enrichment based on all the DEGs identified significant overrepresentation of flavonoid biosynthesis, Cutin/wax biosynthesis, alpha-linolenic acid metabolism (jasmonic acid synthesis), and starch/sucrose metabolism pathways. Weighted gene co-expression network analysis (WGCNA) identified candidate hub genes, including *Zm00001d006069*, *Zm00001d043607* (*hexokinase6*), and *Zm00001d044122* (*anthocyaninless1*), potentially associated with regulatory roles. qRT-PCR validated the differential expression of key genes. Subcellular localization assays further showed that *Zm00001d044122* (*anthocyaninless1*) and *Zm00001d006069* were mainly localized to the nucleus and plasma membrane, respectively. Collectively, this study provides a transcriptomic landscape associated with CSR resistance and suggests candidate coordinated physical, chemical, hormonal, and metabolic defense pathways. The identified hub genes are candidate targets for marker-assisted selection and genetic improvement. However, pathway-level interpretations are transcriptome-based hypotheses and require metabolite profiling and functional validation.

## Simple Summary

Corn stalk rot is a destructive soil-borne disease that reduces maize yield and grain quality worldwide. The molecular basis of natural resistance remains poorly understood, which limits breeding. We compared gene activity in leaves of a resistant and a susceptible maize variety exposed to the disease-causing fungus. The resistant variety kept generally lower background gene activity but, when challenged, coordinated several defense processes. These included production of protective plant compounds called flavonoids, strengthening of the waxy outer surface, activation of a defense hormone called jasmonic acid, and reprogramming of sugar metabolism to supply energy. We identified several candidate regulatory genes potentially associated with these responses, including one involved in sensing sugars and one in flavonoid production. These proteins were found in different parts of the cell, consistent with distinct roles. Our findings suggest candidate coordinated layers of defense against stalk rot. The candidate genes may be potential targets for breeding maize with stronger resistance, which could reduce crop losses and support sustainable agriculture.

## 1. Introduction

Corn stalk rot (CSR), primarily caused by pathogenic fungi such as *Fusarium graminearum*, *F. verticillioides* and *F. proliferatum*, is a devastating soil-borne disease that severely impacts maize yield and grain quality worldwide [1]. The progression of CSR is characterized by the degradation of stalk tissues, which disrupts nutrient transport and often leads to premature plant death [2]. With the increasing frequency of this disease under intensive cropping systems, the development and cultivation of resistant maize varieties have become a crucial strategy for sustainable disease management [3]. However, the molecular mechanisms underlying resistance to CSR remain insufficiently understood, particularly regarding the transcriptional reprogramming that distinguishes resistant from susceptible genotypes during pathogen challenge.

Transcriptome sequencing has emerged as a powerful tool to elucidate the complex regulatory networks involved in plant defense responses [4]. While previous studies have identified individual resistance genes or pathways associated with Fusarium resistance in maize, a comprehensive view of the global transcriptional landscape distinguishing constitutively resistant and susceptible lines is still lacking [5]. In particular, the differential expression patterns of genes involved in secondary metabolism, hormone signaling, and physical barrier formation have not been systematically characterized in the context of CSR resistance. Such information is critical for dissecting the multigenic nature of resistance and for identifying potential targets for molecular breeding.

Over the past two decades, significant efforts have been made to identify genetic determinants underlying maize resistance to stalk rot [6]. Quantitative trait locus (QTL) mapping and genome-wide association studies (GWAS) have revealed multiple genomic regions associated with Fusarium resistance, such as *qRfg1*, *qRfg2*, and a key genomic region on chromosome, highlighting the polygenic nature of this trait [7]. Among the functionally characterized genes, those involved in cell wall reinforcement, such as *Zm00001d037343* (*B6TL27*) and *Zm00001d025833* (*IDP1639*), which participate in Cutin, suberine, and wax biosynthesis, have been shown to contribute to physical barriers against pathogen invasion [8]. Additionally, genes in the flavonoid biosynthesis pathway, including *Zm00001d039833* and *Zm00001d043174*, play critical roles in the production of phytoalexins with direct antifungal activity [9]. The alpha-Linolenic acid metabolism pathway, which governs jasmonic acid (JA) biosynthesis, has also emerged as a key regulator of induced systemic resistance, with genes such as *Zm00001d041204* (*lipoxygenase12*) acting as potential hubs in defense signaling networks [10]. Furthermore, Starch and sucrose metabolism-related genes, including *Zm00001d043607* (*hexokinase6*), are thought to modulate energy availability and sugar signaling, thereby influencing the expression of defense-related genes [11]. Despite these advances, the majority of studies have focused on individual pathways or single genes, and a comprehensive transcriptome-wide comparison between constitutively resistant and susceptible genotypes under uniform field conditions remains limited. Consequently, the coordinated expression patterns that distinguish resistant from susceptible lines are still not fully elucidated.

In this study, we conducted comparative transcriptome analysis of two maize cultivars with contrasting susceptibility to CSR: the CSR-resistant maize cultivar Qi319 (JR) and the CSR-susceptible maize cultivar Ye478 (JS). Leaf samples from both genotypes were collected under identical field conditions and subjected to high-throughput RNA sequencing. Our analysis identified distinct expression profiles between JR and JS, with a total of 6009 differentially expressed genes (DEGs) identified, including genes associated with flavonoid biosynthesis, Cutin and wax metabolism, and alpha-Linolenic acid metabolism. Notably, pathway enrichment and weighted gene co-expression network analysis (WGCNA) identified several candidate hub genes potentially associated with coordination of the defense response.

## 2. Materials and Methods

### 2.1. CSR-Resistant/Susceptible Maize Varieties

According to previous studies, Qi319 (JR) showed high resistance to *Fusarium* CSR, *Pythium* CSR, resistant to corn stalk rot and northern leaf blight. On the contrary, Ye478 (JS) was susceptible to these four maize diseases. JR and JS were selected for our experimental material. In brief, the seeds of the two maize varieties were surface-sterilized with 1% sodium hypochlorite (Sinopharm Chemical Reagent Co., Ltd., Shanghai, China) for 1 min, and then washed three times with sterile water. The seeds were then sown in a sterilized mixed substrate (local supplier, Fuzhou, China) (vermiculite:humus = 1:1) and cultivated in a greenhouse under conditions of 25–28 °C, 60–70% relative humidity, and a photoperiod of 14 h/10 h (light/dark). When the seedlings of both maize varieties reached the 4- to 5-leaf stage, two fully expanded leaves were randomly collected from three individual plants of each variety and rapidly pooled to form a single sample. The samples were frozen in liquid nitrogen and subsequently stored at –80 °C. Three biological replicates were prepared for each maize variety.

### 2.2. RNA Extraction and Transcriptome Sequencing

Total RNA of each sample was isolated from the maize leaves using the TRlzol Reagent (Life Technologies, Carlsbad, CA, USA) following the protocol manual. RNA integrity and concentration were subsequently evaluated using an Agilent 2100 Bioanalyzer (Agilent Technologies, Inc., Santa Clara, CA, USA). The mRNA was isolated using NEBNext Poly(A) mRNA Magnetic Isolation Module (NEB, E7490). The cDNA library was prepared according to the manufacturer’s instructions for the NEBNext Ultra RNA Library Prep Kit (Catalog No. E7760S, New England Biolabs, Ipswich, MA, USA) and NEBNext Multiplex Oligos (New England BioLabs, #E7355L) for Illumina (E7500, New England Biolabs, Ipswich, MA, USA). In brief, the enriched mRNA was fragmented into approximately 200nt RNA inserts, which served as a template for the first-strand cDNA and the second cDNA synthesis. The double-stranded cDNA was subjected to end-repair/dA-tail and adaptor ligation. The suitable fragments were isolated using the Agencourt AMPure XP beads (Beckman Coulter, Inc., Brea, CA, USA) and enriched by PCR amplification. Finally, the constructed cDNA libraries derived from the maize were sequenced on a flow cell using an Illumina HiSeq™ sequencing platform (Illumina, Inc., San Diego, CA, USA).

### 2.3. Bioinformatic Analysis of Transcriptome Sequencing Data

Low-quality reads, such as those containing only adaptor, unknown nucleotides >5%, or those in which more than 20% of bases had a Phred quality score < Q20 (i.e., sequencing error rate > 1%), were removed using a Perl (version 5.40.2) script. The clean reads, filtered from the raw reads, were mapped to the B73 maize genome (v5.0) using TopHat2 software (version 2.1.1) [12]. The aligned records from the aligners in BAM/SAM format were further examined to remove potential duplicate molecules. Gene expression levels were estimated using FPKM values (fragments per kilobase of exon per million fragments mapped) by the Cufflinks software (version 2.2.1) [13]. DESeq (version 1.48.0) was employed to evaluate differential gene expression between JR and JS [14]. Here, only genes with an absolute value of log2 ratio ≥2 and a *p*-value significance score <0.05 were used for subsequent analysis. Genes were compared against several protein databases by BLASTX. These databases, including the NCBI non-redundant protein (Nr) database and the Swiss-Prot database, were searched with a cut-off E-value of 10^−5^. Additionally, genes were searched against the NCBI non-redundant nucleotide sequence (Nt) database using BLASTn with a cut-off E-value of 10^−5^. Genes were retrieved based on the best BLAST hit (highest score) along with their protein functional annotation. Gene Ontology (GO) terms were assigned based on the Nr BLAST results, which were imported into the Blast2GO program [15]. GO annotations for the genes were obtained through Blast2GO, which mapped all annotated genes to GO terms in the database and counted the number of genes associated with each term. A Perl script was then used to plot the GO functional classification for the unigenes with GO term hits, providing an overview of the distribution of gene functions. The obtained annotations were further enriched and refined using TopGO (R package) (version 2.60.0) [16]. The gene sequences were also aligned to the Clusters of Orthologous Groups (COG) database to predict and classify functions. KEGG pathways were assigned to the assembled sequences by a Perl script. To explore co-expression modules and candidate hub genes potentially associated with CSR resistance, weighted gene co-expression network analysis (WGCNA) was performed with the R package WGCNA (version 1.73) based on the expression matrix of all differentially expressed genes (DEGs). Genes with low expression were filtered by retaining genes with FPKM > 1 in at least three samples.

### 2.4. Quantitative Real-Time PCR (qRT-PCR) Analysis

The expression of eight hub genes was confirmed by qRT-PCR. JR and JS were employed as the experimental materials. Briefly, these two cultivars were planted in a greenhouse at the Fujian Academy of Agricultural Science. The method for sample preparation followed the procedure described in Section 2.1. Total RNA was extracted using the Pure Tissue RNA Kit (Cwbiotech, Beijing, China) and assessed for quality via agarose gel electrophoresis. Up to 1 μg of total RNA was used to synthesize cDNA with the TUREscript 1st Strand cDNA SYNTHESIS Kit. A 20 μL reaction volume was prepared for qRT-PCR, containing 5 μL of diluted cDNA template, 0.5 μL of each specific primer, 4 μL of 5 × RT Reaction Mix, 0.8 μL of TUREscript H- RTase, and 9.7 μL of ddH_2_O. The PCR cycling conditions consisted of an initial step at 45 °C for 40 min, followed by 65 °C for 10 min. *β-tubulin* was used as a stable reference gene. The primer pairs specific to each gene for qRT-PCR were designed using NCBI Primer-BLAST and are listed in Table S1. To ensure the accuracy of the results, three biological replicates were performed. The relative expression levels of the target genes were calculated using the 2^−ΔΔ^Ct method [1]. Statistical differences in gene expression between CSR-resistant and control groups were assessed using a two-tailed unpaired Wilcoxon rank-sum test at a threshold *p*-value  <  0.05.

### 2.5. Subcellular Localization of Candidate Proteins

The coding regions of *Zm00001d044122* (*anthocyaninless1*) and *Zm00001d006069* were separately cloned into the pBWA(V)H2STMVQ vector to generate eGFP fusion constructs. The 35S:eGFP empty vector was used as a control. These constructs were transiently expressed in *N. benthamiana* leaves via *Agrobacterium tumefaciens* GV3101 (psoup-p19). Briefly, Agrobacterium cultures were grown in YEB medium (Solarbio, Beijing, China), collected by centrifugation at 4000 rpm for 5 min, and resuspended in 10 mM MgCl_2_ (Sinopharm Chemical Reagent Co., Ltd., Shanghai, China) containing 120 μM acetosyringone (Solarbio, Beijing, China) to an OD_600_ of approximately 0.6. After incubation at 28 °C for at least 1 h or at 4 °C for at least 3 h, the suspensions were infiltrated into the abaxial side of fully expanded tobacco leaves using a needleless 1 mL syringe. After low-light incubation for 2–4 days, leaf samples were examined using a Nikon C2-ER confocal laser-scanning microscope (Nikon, Tokyo, Japan). GFP fluorescence was excited at 488 nm and detected at 510 nm. Chlorophyll autofluorescence was excited at 488/640 nm and detected at 680 nm. Images were collected for the GFP, chlorophyll channel, bright field, and merged channels. At least three independent leaf samples were observed.

## 3. Results

### 3.1. Transcriptomics Basic Data of CSR-Resistant/Susceptible Maize Samples

To investigate the differences in transcriptional levels, leaf samples from JR and JS were collected. According to the transcriptome sequencing data presented in Table 1, the JR and JS leaf samples were subjected to read mapping analysis. The results showed that the mapping rates of all six samples to the maize reference genome exceeded 87%. Specifically, the total mapping rates for JR samples ranged from 88.16% to 89.02%, while those for JS samples ranged from 87.28% to 88.94%. The uniquely mapped reads accounted for a high proportion in all samples, ranging from 80.60% to 82.14% in JR samples and from 79.04% to 83.20% in JS samples. Notably, the proportion of spliced reads was slightly higher in JR samples (32.33–33.36%) than in JS samples (26.10–29.73%), whereas the proportion of non-spliced reads was slightly higher in JS samples. Overall, differences in mapping characteristics were observed between resistant and susceptible varieties, particularly in the proportion of spliced reads, suggesting potential variation in gene structure or expression regulation between the two groups.

A total of 33,162 genes were expressed at detectable levels. Based on their FPKM values, these genes were assigned to different expression-level bins, and the number of genes in each bin was counted (Table S2). In the JR group, the proportion of low-expression genes (FPKM < 1) ranged from 45.72% to 50.08%, which was higher than that in the JS group (41.77–45.90%). Conversely, in the moderate-to-high expression intervals (FPKM > 10), the JS group exhibited a higher proportion of genes (21.08–25.86%) compared with the JR group (19.73–23.69%). Notably, in the 10 < FPKM < 30 interval, JS1 and JS3 showed gene proportions of 15.45% and 14.32%, respectively, markedly higher than those observed in JR samples (10.58–12.03%). Additionally, in the high-expression interval (FPKM > 50), JS samples also displayed a higher proportion of genes (5.10–5.57%) than JR samples (4.14–4.99%). These results suggest that susceptible varieties harbor more genes expressed at moderate-to-high levels, whereas resistant varieties accumulate more low-expression genes, potentially reflecting differences in basal transcriptional activity or expression regulation of stress-responsive genes between the two groups.

### 3.2. Overall Expression Differences in Genes Between CSR-Resistant/Susceptible Maize Samples

**Figure 1:**
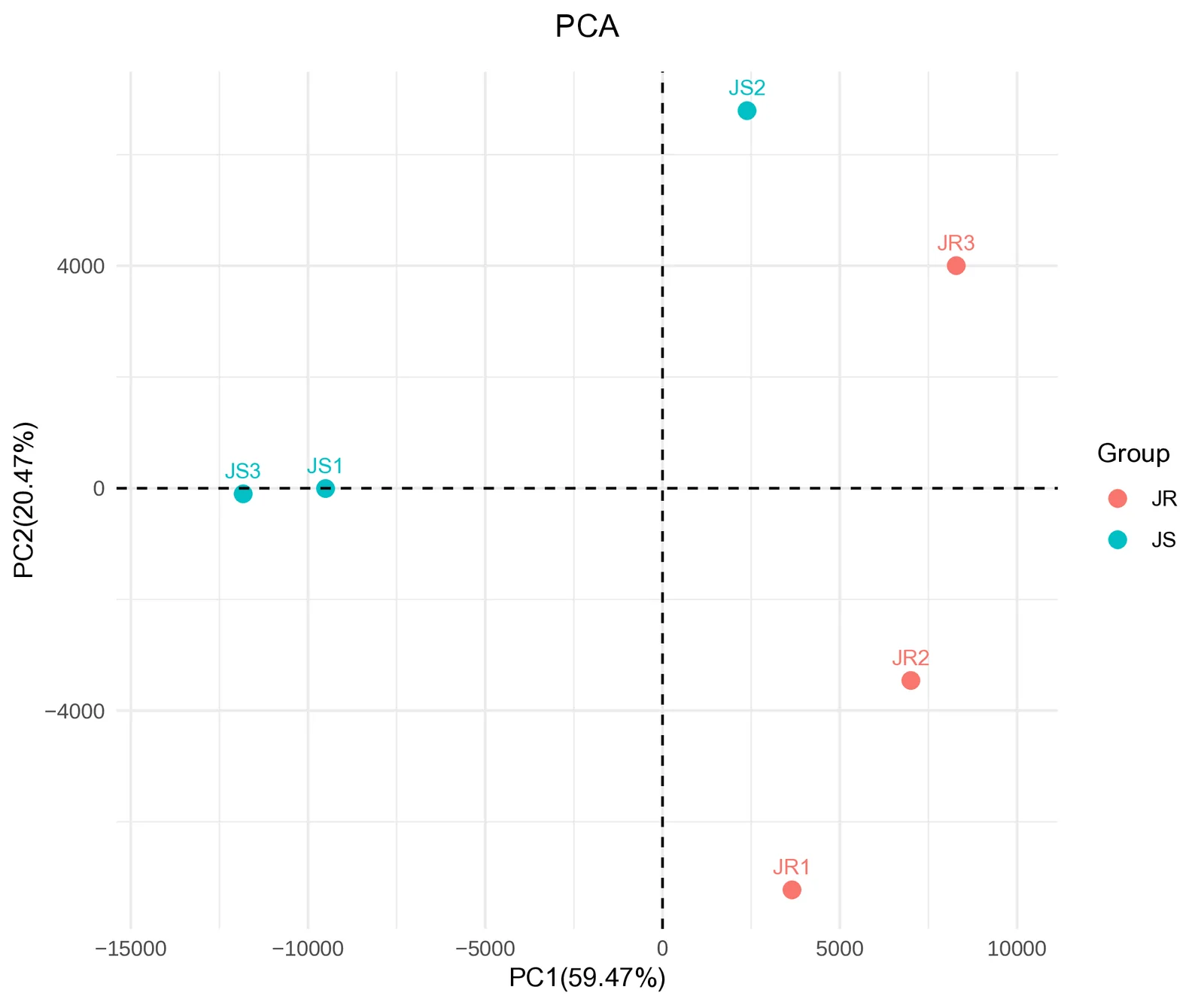
Principal component analysis (PCA) of gene expression between CSR-resistant/susceptible maize samples based on Bray–Curtis distances. Significant differences among the plant compartments were determined using PERMANOVA (*n*  =  6).

Principal component analysis (PCA) was employed to assess the overall expression differences in genes between JR and JS samples. The PCA results showed that JR samples were significantly separated from the JS samples (Figure 1). Among them, the first principal component explained 59.47% of the total variance, and the second principal component explained 20.47% of the total variance, amounting to 79.94%. These results indicate that the overall gene expression patterns differed significantly between JR and JS samples.

Based on the FPKM-level expression data, differential expression analysis of genes was conducted using DESeq2. *p*-value less than 0.05 and |log2(foldchange)| greater than 1 were set as the standard for screening differentially expressed genes (DEGs). A total of 6009 DEGs were found between CSR-resistant/susceptible maize samples, including 3074 up-regulated and 2935 down-regulated DEGs.

**Figure 2:**
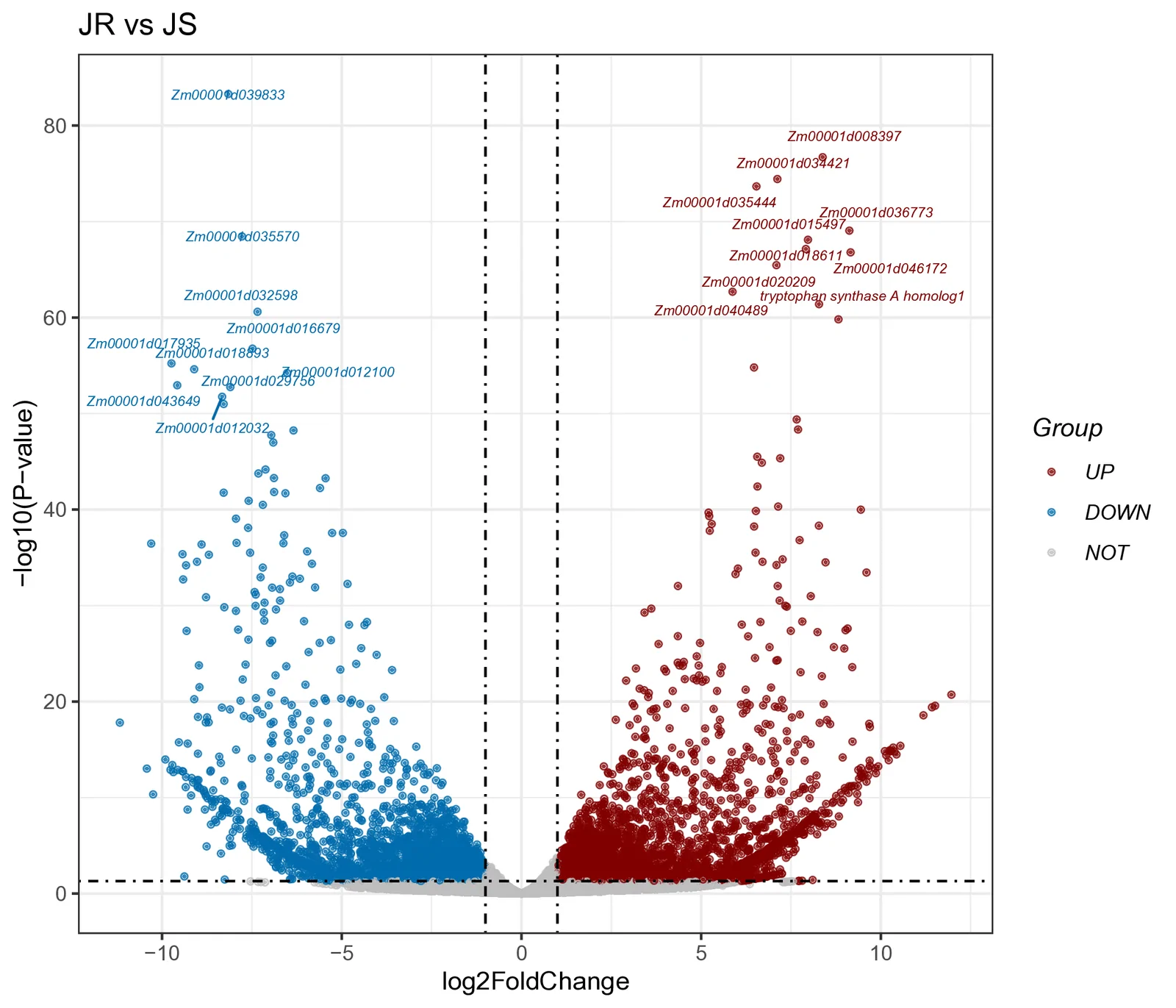
Volcano plot of differential gene expression distribution. *p*-value less than 0.05 and |log2(foldchange)| greater than 1 were set as the standard for screening. The up-regulated DEGs were marked by red circles and down-regulated DEGs were marked by blue circles.

The volcano plot (Figure 2) showed the top 10 DEGs according to the *p*-values. Among them, *Zm00001d008397*, *Zm00001d034421*, *Zm00001d035444*, *Zm00001d036773*, *Zm00001d015497* (*aco6*), *Zm00001d018611* (*tps31*), *Zm00001d046172*, *Zm00001d020209*, *Zm00001d040489* (*thx57*) and *Zm00001d034460* (*igl1*) were the top 10 up-regulation DEGs. Meanwhile, *Zm00001d039833*, *Zm00001d035570*, *Zm00001d032598* (*IDP174*), *Zm00001d016679*, *Zm00001d017935*, *Zm00001d018893*, *Zm00001d012100*, *Zm00001d043649*, *Zm00001d029756* and *Zm00001d012032* were the top 10 down-regulation DEGs.

### 3.3. Differences in KEGG Functional Pathways Between CSR-Resistant/Susceptible Maize Samples

**Figure 3:**
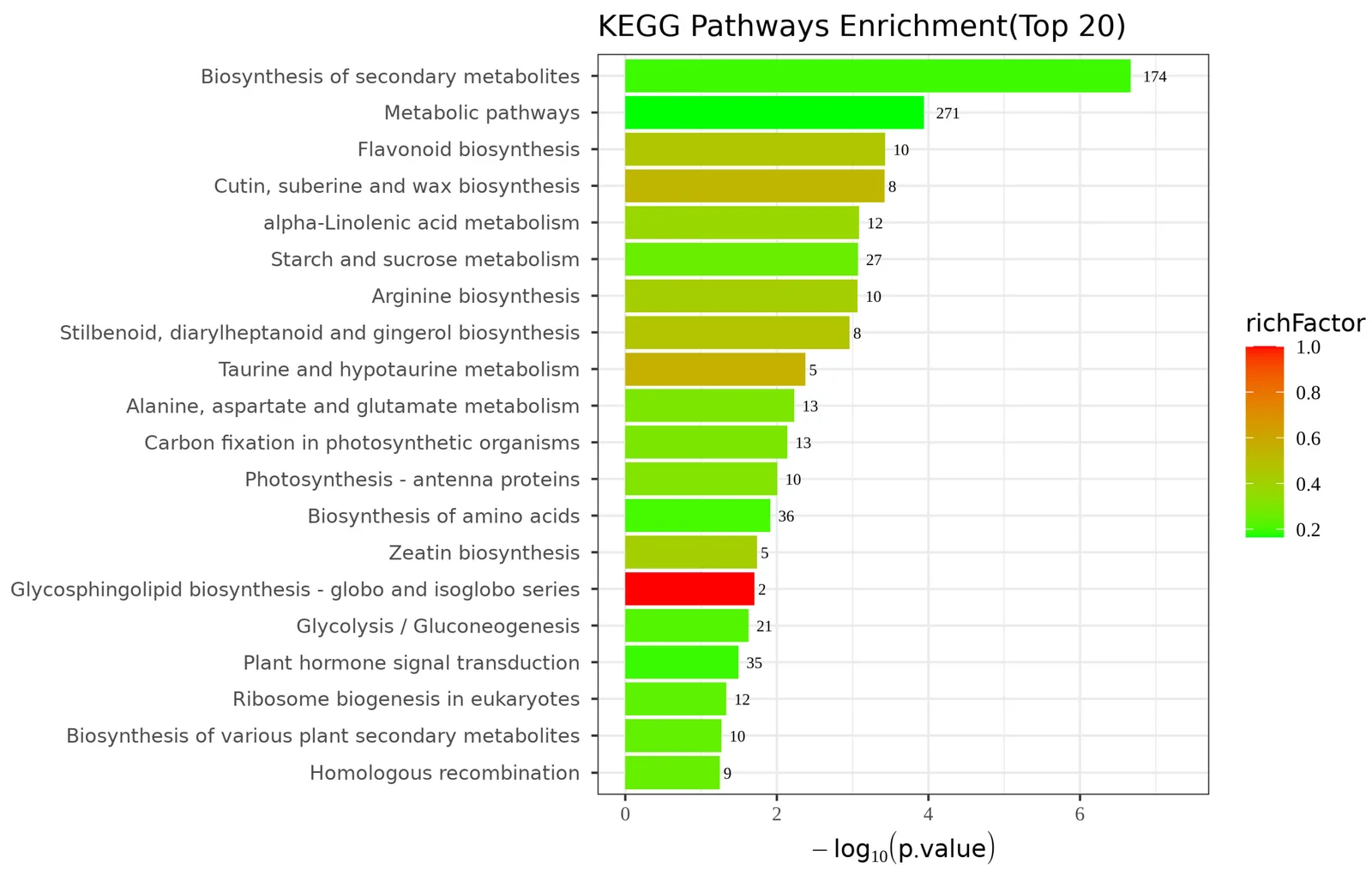
KEGG enrichment bar chart of differentially expressed genes. The number of DEGs is shown to the right of terms.

To investigate the functional pathways involving the DEGs, KEGG annotation was performed. As shown in Figure 3, Biosynthesis of secondary metabolites, Metabolic pathways, Cutin, suberine and wax biosynthesis, Flavonoid biosynthesis, Arginine biosynthesis, Starch and sucrose metabolism, alpha-Linolenic acid metabolism, and Stilbenoid, diarylheptanoid and gingerol biosynthesis pathways were significantly enriched (FDR < 0.05). A total of 174 and 271 DEGs were involved in Biosynthesis of secondary metabolites and Metabolic pathways, respectively. Since these two pathways cover a very wide range and are generally regarded as the primary metabolism and the general background, lacking specificity; therefore, in the subsequent analysis, we no longer focus on them in order to concentrate on the more detailed pathways that can better reflect the specific changes in the sample phenotype. A total of 10, 8, 27 and 12 DEGs were enriched in Flavonoid biosynthesis, Cutin, suberine and wax biosynthesis, Starch and sucrose metabolism and alpha-Linolenic acid metabolism pathways. Flavonoids are important phytoalexins in corn, possessing direct antibacterial activity and participating in lignin synthesis to strengthen the cell wall. They are one of the most crucial secondary metabolic pathways in the disease resistance response. Cutin, suberine and wax biosynthesis directly contribute to the formation of the physical barrier on the surface of corn. The metabolism of Starch and sucrose regulates the dynamic distribution of soluble sugars, providing energy for disease resistance and also inducing the expression of defense genes through sugar signaling. Alpha-Linolenic acid metabolism is the direct synthesis pathway of jasmonic acid (JA). JA, as the core defense hormone, regulates the systemic resistance of corn against stem rot, leaf spot diseases, and pest feeding. These four pathways respectively correspond to barrier reinforcement in disease resistance, synthesis of antibacterial substances, reprogramming of energy metabolism, and activation of hormone signaling. These four pathways are candidate entry points for generating transcriptome-based hypotheses about maize CSR resistance.

**Figure 4:**
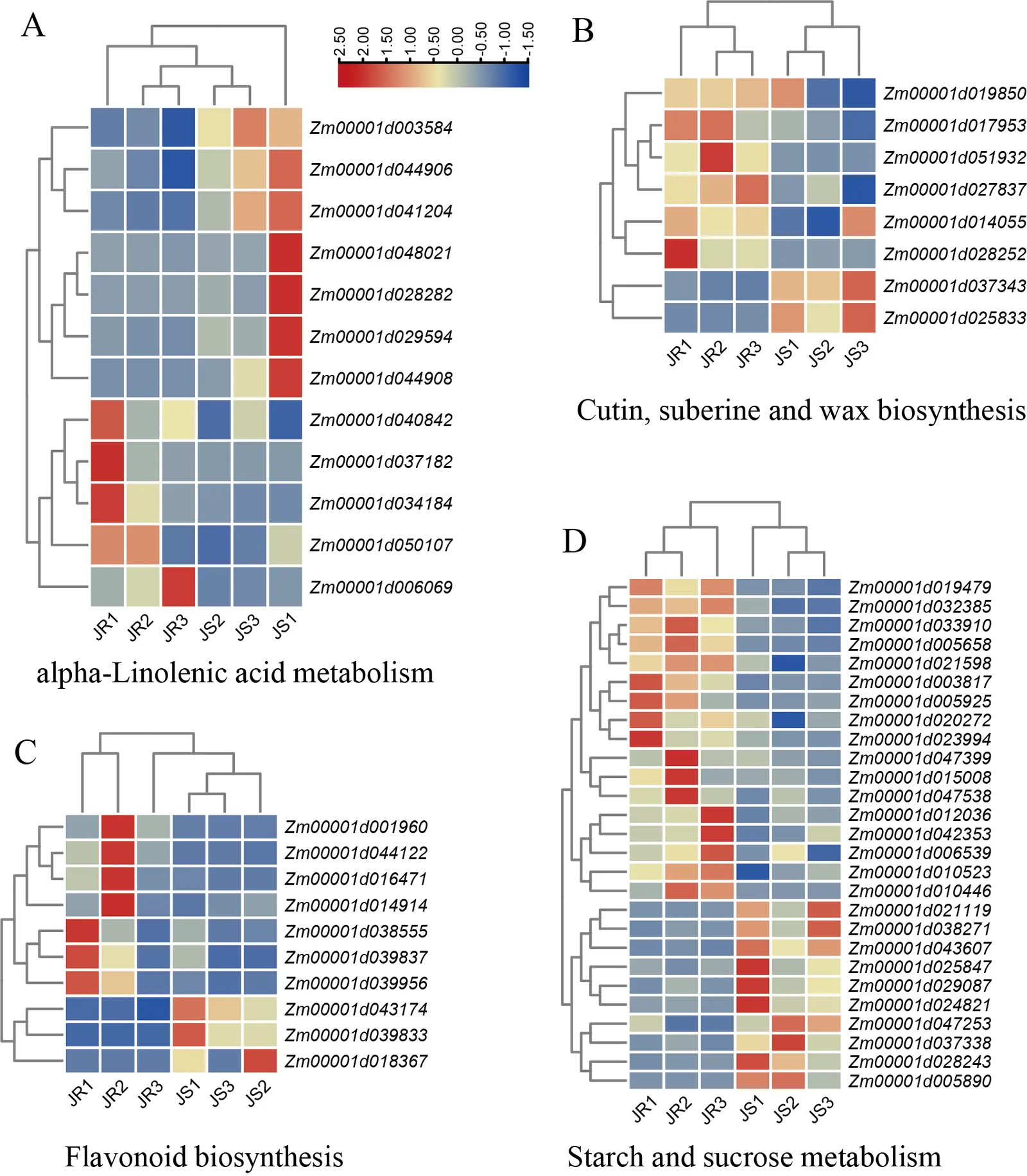
Heatmap showing the expression trends of DEGs involved in alpha-Linolenic acid metabolism (**A**), Cutin, suberine and wax biosynthesis (**B**), Flavonoid biosynthesis (**C**) and Starch and sucrose metabolism (**D**) KEGG terms. The up-regulated DEGs were marked in red, and the down-regulated DEGs were marked in blue.

The expression heatmap showed that the expression levels of *Zm00001d003584* (*12-oxo-phytodienoic acid reductase5*), *Zm00001d044906* (*12-oxo-phytodienoic acid reductase2*) and *Zm00001d041204* (*lipoxygenase12*) were consistently up-regulated across all the CSR-resistant maize samples (Figure 4A). In the Cutin, suberine and wax biosynthesis pathway, *Zm00001d037343* (*B6TL27*) and *Zm00001d025833* (*IDP1639*) showed a similar expression pattern (Figure 4B). In the Flavonoid biosynthesis pathway, the pathogen also enhanced the expression of *Zm00001d039833* and *Zm00001d043174 (umc2381*). A total of ten DEGs, like *Zm00001d021119* (*glu14*), *Zm00001d038271* and *Zm00001d047253* (*sus1*), were up-regulated in Starch and sucrose metabolism.

### 3.4. WGCNA Analysis Based on the Expression Level of DEGs

**Figure 5:**
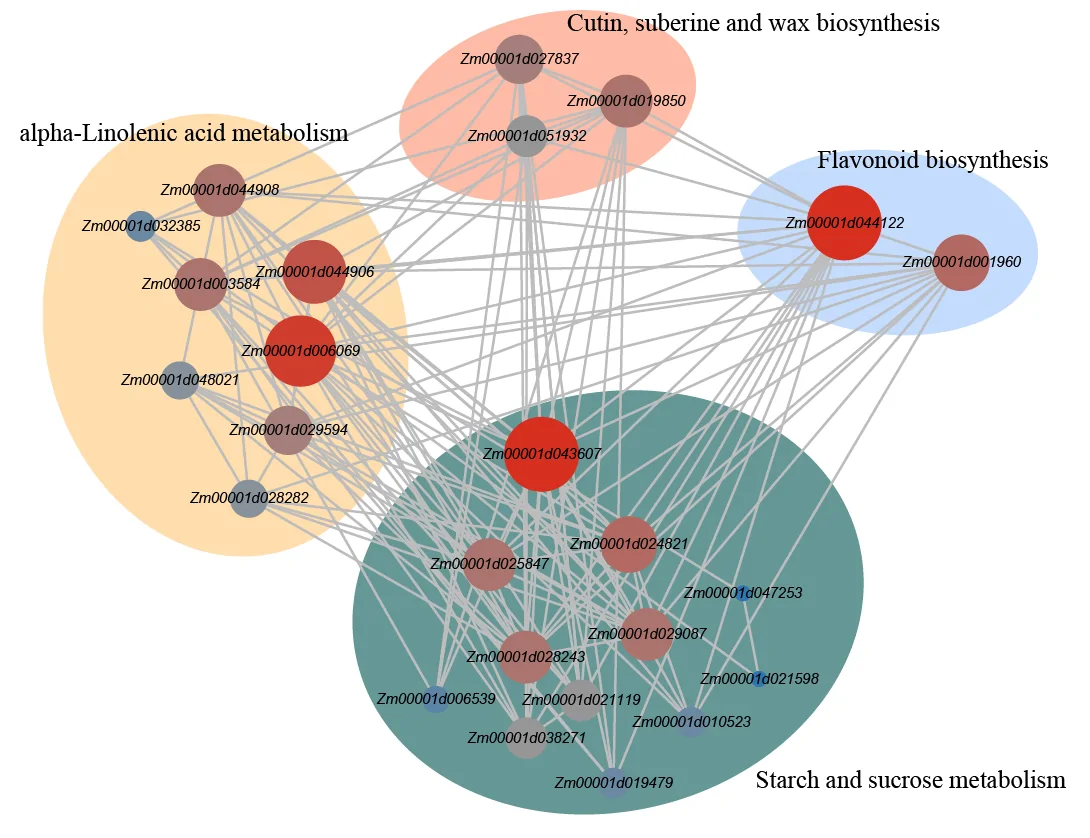
Co-expression network of the DEGs involved in alpha-Linolenic acid metabolism, Cutin, suberine and wax biosynthesis, Flavonoid biosynthesis and Starch and sucrose metabolism KEGG terms. Each node represents a gene, and edges represent weighted correlations. Node size and color are proportional to their connectivity, with larger nodes indicating hub genes.

To identify co-expressed gene modules associated with CSR and to explore the underlying network-level regulatory mechanisms, we performed WGCNA (version 1.73) on the transcriptome dataset. A gene co-expression network within the turquoise module was constructed using Cytoscape (v3.9.1). The network visualized the interaction landscape of the top 25 hub genes, with nodes representing genes and edges representing weighted correlations (Figure 5). Notably, *Zm00001d006069* (alpha-Linolenic acid metabolism, log2FC = 3.39, *p*-value = 0.002), *Zm00001d043607* (*hexokinase6*, Starch and sucrose metabolism, log2FC = −1.90, *p*-value = 7.35 × 10^−7^) and *Zm00001d044122* (*anthocyaninless1*, Flavonoid biosynthesis, log2FC = 5.95, *p*-value = 8.20 × 10^−14^) emerged as the nodes with the highest degree centrality, suggesting they are candidate hub genes potentially associated with regulatory roles.

### 3.5. qRT-PCR of the Hub Genes in CSR-Resistant Maize

**Figure 6:**
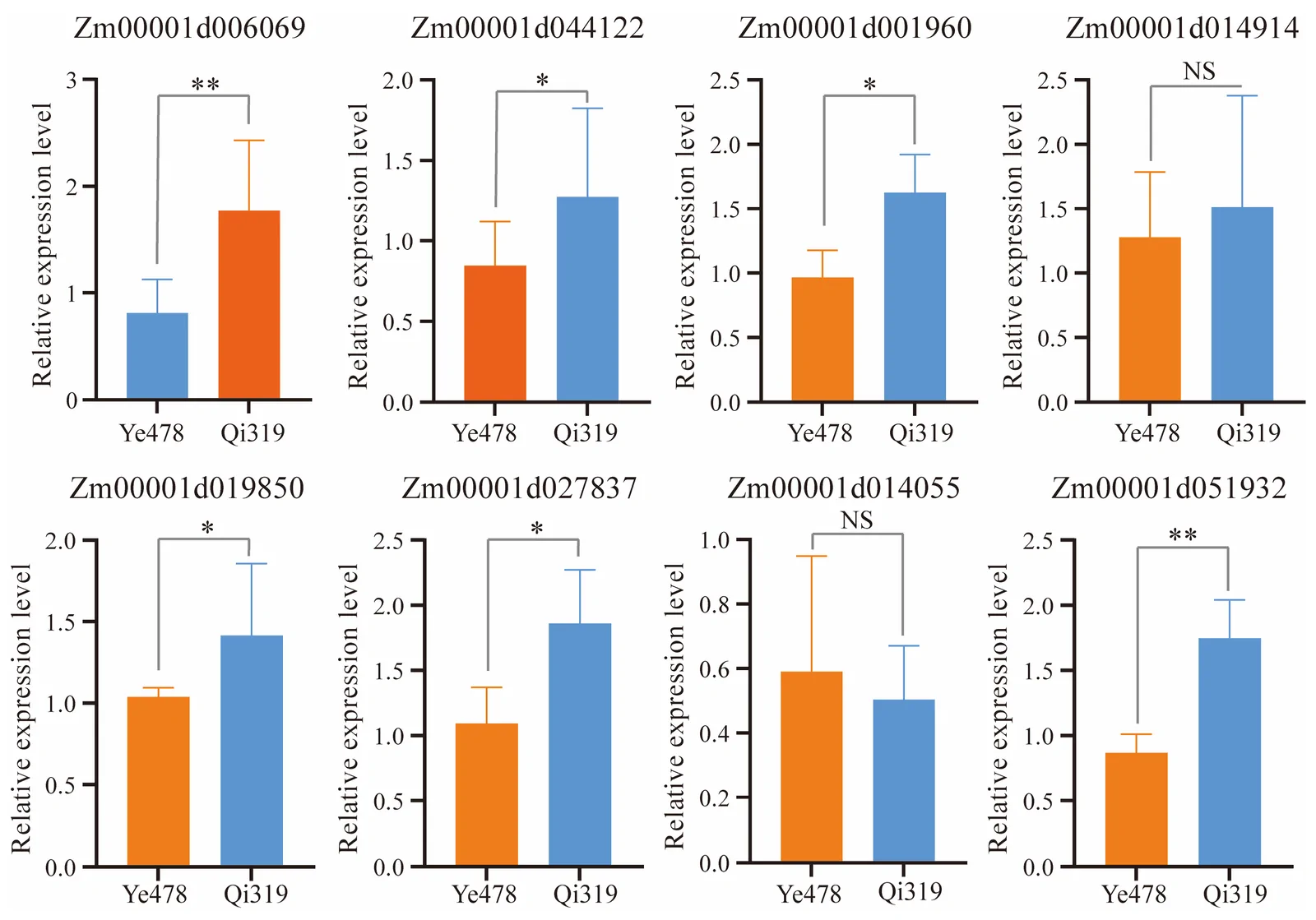
Relative expression levels of eight hub genes determined by qRT-PCR in CSR-resistant cultivar Qi319 and CSR-susceptible cultivar Ye478. Significant differences were determined by the Wilcoxon rank-sum test (* *p* < 0.05; ** *p* < 0.01; NS *p* > 0.05).

To validate the accuracy of transcriptome data, eight DEGs associated with alpha-Linolenic acid metabolism (*Zm00001d006069*), Flavonoid biosynthesis (*Zm00001d044122* (*anthocyaninless1*), *Zm00001d001960* (*fht1*) and *Zm00001d014914* (*a2*)) and Cutin, suberine and wax biosynthesis (*Zm00001d019850*, *Zm00001d027837* (*ms26*), *Zm00001d014055* (*cl3929_1*) and *Zm00001d051932*) pathways were selected for qRT-PCR. Results showed that the expression levels of *Zm00001d006069*, *Zm00001d044122* (*anthocyaninless1*), *Zm00001d001960* (*fht1*), *Zm00001d019850*, *Zm00001d027837* (*ms26*) and *Zm00001d051932* were significantly promoted in JR (Figure 6). These results were consistent with the transcriptome data. At the same time, we also noticed that *Zm00001d014914* (*a2*), which is associated with flavonoid biosynthesis, did not show statistically significant expression differences; this is consistent with the overall expression trend of the transcriptome. The expression level of *Zm00001d014055* (*cl3929_1*) was not consistent between the transcriptome and qRT-PCR results, suggesting that *Zm00001d014055* (*cl3929_1*) may have no relation to maize CSR resistance.

### 3.6. Subcellular Localization of Zm00001d044122 and Zm00001d006069

**Figure 7:**
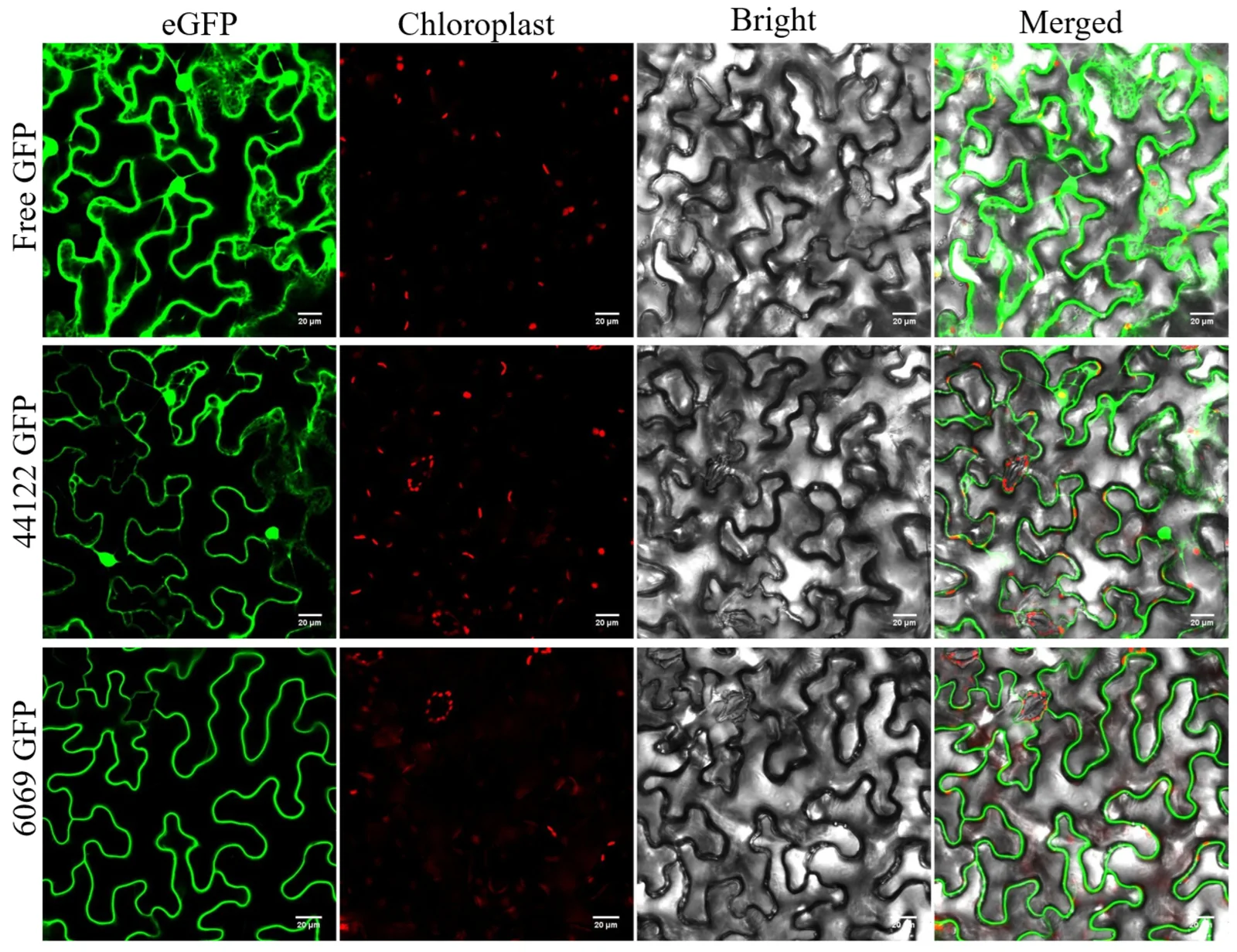
Subcellular localization of *Zm00001d044122* and *Zm00001d006069* in *N. benthamiana* epidermal cells. For each construct, images from left to right show the GFP channel, chloroplast autofluorescence, bright-field, and merged image. Scale bars = 20 μm.

To further investigate the potential functions of the hub genes, we examined the subcellular localization of *Zm00001d044122* (*anthocyaninless1*) and *Zm00001d006069* in *N. benthamiana* epidermal cells (Figure 7). The 35S:eGFP empty vector produced fluorescence in the cytoplasm and at the cell periphery. For the Zm00001d044122-eGFP fusion, strong GFP signals were predominantly detected in the nucleus, with weaker signals in the cytoplasm, indicating that *Zm00001d044122* is mainly localized to the nucleus. In contrast, the Zm00001d006069-eGFP fusion showed fluorescence mainly at the cell periphery, consistent with plasma membrane localization. The chlorophyll autofluorescence was clearly separated from the GFP signal, excluding chloroplast localization. These results suggest that *Zm00001d044122* and *Zm00001d006069* may function in different subcellular compartments, possibly involving nuclear transcriptional regulation and membrane-associated defense signaling, respectively.

## 4. Discussion

In this study, comparative transcriptome analysis between the CSR-resistant cultivar Qi319 (JR) and the CSR-susceptible cultivar Ye478 (JS) revealed extensive transcriptional reprogramming underlying constitutive resistance. A total of 6009 differentially expressed genes (DEGs) were identified, with 3074 upregulated and 2935 downregulated in JR, indicating a nearly balanced but highly coordinated transcriptional shift. Notably, the resistant variety exhibited a higher proportion of low-expression genes (FPKM < 1), whereas the susceptible variety displayed more genes in the moderate-to-high expression range (FPKM > 10 and > 50), suggesting that JR maintains a more stringent basal transcriptional regime [17]. This pattern may reflect an adaptive strategy to avoid the constitutive expression of defense-related genes, which often impose growth and fitness costs, while preserving the capacity for rapid induction upon pathogen attack [18]. In contrast, the globally elevated baseline in JS could lead to metabolic inefficiency or reduced responsiveness to infection, a phenomenon observed in other pathosystems where susceptible genotypes exhibit “transcriptional noise” or impaired defense priming [19]. Principal component analysis strongly separated JR from JS along the first principal component (59.47% variance), confirming that the resistance-associated transcriptional architecture is highly distinct. Among the top upregulated DEGs in JR, genes such as *Zm00001d008397* and *Zm00001d034421* represent novel candidates for further functional characterization; *Zm00001d008397* encodes a protein homologous to the Arabidopsis salt tolerance-related protein (AT1G13930) and may be involved in ABA-responsive gene expression, but its role in disease resistance has not been previously reported. *Zm00001d034421* lacks any known functional annotation in public databases and was only observed to be differentially expressed upon fungal inoculation. Their high expression specifically in the resistant genotype, together with their absence from the major enriched defense pathways (Figure 3), suggests that they may represent previously unrecognized components of the CSR resistance network. Therefore, these two genes are prioritized for future functional validation. The top downregulated genes, including *Zm00001d039833* (flavonoid pathway) and *Zm00001d035570*, suggest that certain secondary metabolism genes are constitutively suppressed in resistant lines, possibly to permit pathogen-triggered inducibility [20].

The discrepancy for *Zm00001d014055* (*cl3929_1*) between RNA-seq and qRT-PCR likely stems from its very low expression (FPKM < 0.5) and modest fold change (≈1.8), both of which challenge reliable detection. Technical factors—such as differences in reverse transcription priming, primer efficiency, or biological variation—may be amplified for low-abundance transcripts. Thus, its role in CSR resistance remains inconclusive and requires validation in stalk tissues with more sensitive methods (e.g., ddPCR) or additional replicates. This case highlights the need for caution when interpreting negative qPCR results for low-expression genes.

Functional enrichment analysis demonstrated that DEGs were significantly overrepresented in four key pathways: flavonoid biosynthesis, Cutin, suberine and wax biosynthesis, alpha-linolenic acid metabolism (jasmonic acid synthesis), and Starch and sucrose metabolism. These pathways correspond to four pillars of plant immunity—antimicrobial phytoalexin production, physical barrier reinforcement, hormone signaling, and metabolic reprogramming, respectively. In the flavonoid biosynthesis pathway, genes such as *Zm00001d039833* and *Zm00001d043174* were downregulated in JR under non-challenged conditions, consistent with a priming model wherein phytoalexin biosynthesis genes are maintained at low basal levels but are poised for strong induction upon pathogen perception [21]. Conversely, in the Cutin, suberine and wax biosynthesis pathway, genes like *Zm00001d037343* (*B6TL27*) and *Zm00001d025833* (*IDP1639*) showed expression patterns suggestive of enhanced constitutive physical barrier deposition in JR. This aligned with previous reports that cell wall reinforcement and cuticular wax accumulation are critical for resistance against soil-borne *Fusarium* species [22]. The alpha-linolenic acid metabolism pathway, which directly supplies substrates for jasmonic acid (JA) biosynthesis, was consistently upregulated in JR, with *Zm00001d041204* (*lipoxygenase12*) and *Zm00001d006069* showing elevated expression. JA is a master regulator of induced systemic resistance and has been shown to mediate maize defense against stalk rot [23]. The consistent upregulation of these genes across JR samples suggests that JA signaling is constitutively enhanced in the resistant genotype, a feature that likely contributes to rapid defense activation. Additionally, Starch and sucrose metabolism-related genes, including *Zm00001d043607* (*hexokinase6*) and *Zm00001d021119,* were upregulated in JR, indicating a reprogramming of carbon flux and energy allocation to support sustained defense responses. Sugars not only fuel metabolic demands but also act as signaling molecules that modulate defense gene expression [24]. Collectively, these pathway-level changes suggest that CSR resistance in JR may involve multiple candidate defense layers rather than a single mechanism; this hypothesis requires metabolite profiling and functional validation.

Weighted gene co-expression network analysis (WGCNA) further identified co-expression modules and candidate hub genes potentially associated with the regulatory architecture of CSR resistance. Among these, *Zm00001d006069* (alpha-linolenic acid metabolism), *Zm00001d043607* (*hexokinase6*, Starch and sucrose metabolism), and *Zm00001d044122* (*anthocyaninless1*, flavonoid biosynthesis) exhibited the highest degree centrality, suggesting they are candidate hub genes potentially associated with coordination of downstream targets across different functional pathways. The identification of *Zm00001d006069* as a hub is particularly intriguing, as it encodes a putative lipoxygenase or related enzyme in the JA biosynthesis cascade, consistent with the observed enrichment of JA-related pathways [25]. Similarly, *Zm00001d044122* (*anthocyaninless1*) likely participates in the production of antifungal flavonoids, while *Zm00001d043607* (*hexokinase6*) may modulate sugar signaling and energy status [20,26]. These hub genes represent high-priority targets for further functional validation via mutant analysis or overexpression studies. qRT-PCR validation of eight selected DEGs confirmed the differential expression of most genes, including *Zm00001d006069*, *Zm00001d044122* (*anthocyaninless1*), *Zm00001d001960* (*fht1*), *Zm00001d019850*, *Zm00001d027837* (*ms26*), and *Zm00001d051932*, showing excellent concordance with RNA-seq data. Only *Zm00001d014055* (*cl3929_1*) yielded discordant results, suggesting it may not be directly involved in CSR resistance, whereas *Zm00001d014914* (*a2*), though not statistically significant, followed the same trend as the transcriptome data. These validation results reinforce the reliability of our transcriptomic analysis and highlight the importance of experimental verification for individual candidate genes.

In addition, subcellular localization assays showed that *Zm00001d044122* (*anthocyaninless1*) was predominantly nuclear, whereas *Zm00001d006069* was mainly localized to the plasma membrane. The nuclear localization of *Zm00001d044122* is consistent with its putative role as a transcriptional regulator in flavonoid biosynthesis, while the membrane localization of *Zm00001d006069* suggests that it may participate in membrane-associated signaling or lipid metabolism during defense. These findings further support the functional divergence of the hub genes and their potential coordination in the CSR resistance network.

Collectively, this study provides a global transcriptomic landscape of constitutive CSR resistance in maize, revealing coordinated regulation of physical barriers, antimicrobial synthesis, JA signaling, and metabolic reprogramming. The identified hub genes, including *Zm00001d006069*, *Zm00001d043607* (*hexokinase6*), and *Zm00001d044122* (*anthocyaninless1*), represent promising targets for marker-assisted selection and genetic improvement of CSR resistance. While further validation in stalk tissues and through functional genetic approaches is needed, our findings significantly advance the understanding of multigenic resistance mechanisms and provide a practical resource for sustainable maize disease management—specifically, candidate markers for marker-assisted selection (e.g., SNPs linked to *Zm00001d006069* and *Zm00001d044122*), targets for CRISPR-Cas9 gene editing (e.g., overexpression or promoter editing of hub genes), and expression-based diagnostic tools for high-throughput resistance phenotyping.

## 5. Conclusions

In summary, this study provides a comprehensive transcriptomic comparison between a constitutively resistant and a susceptible maize cultivar under field conditions. A total of 6009 differentially expressed genes were identified, with the resistant genotype exhibiting a distinct transcriptional architecture characterized by lower basal expression levels and candidate coordinated activation of defense-related pathways. Functional enrichment and WGCNA suggested that CSR resistance is associated with candidate coordinated regulation of four pathways: flavonoid biosynthesis, Cutin, suberine and wax biosynthesis; alpha-linolenic acid metabolism (jasmonic acid synthesis); and Starch and sucrose metabolism. Hub genes such as *Zm00001d006069*, *Zm00001d043607* (*hexokinase6*), and *Zm00001d044122* (*anthocyaninless1*) were identified as central regulators within these pathways and represent promising targets for marker-assisted selection or genetic engineering. Subcellular localization analysis further indicated that *Zm00001d044122* (*anthocyaninless1*) and *Zm00001d006069* were mainly localized to the nucleus and plasma membrane, respectively, supporting their distinct roles in the CSR resistance network. These findings provide candidate pathways/genes, transcriptome-based hypotheses for multigenic CSR resistance, and candidate targets for marker-assisted selection and subsequent functional characterization, supporting the development of maize varieties with improved stalk rot resistance. Although the analysis was restricted to seedling leaf tissues and the functional roles of hub genes remain to be validated in stalk tissues and broader germplasm, these findings provide a foundation for future work.

## Figures and Tables

**Table 1:** Statistics table of reads aligned with the maize reference genome.

Sample Name	Total Reads	Total Mapped	Multiple Mapped	Unique Mapped	Non-Splice Reads	Splice Reads
JR1	45,395,730	40,316,483 (88.81%)	3,027,773 (6.67%)	37,288,710 (82.14%)	22,143,332 (48.78%)	15,145,378 (33.36%)
JR2	46,740,036	41,607,126 (89.02%)	3,800,520 (8.13%)	37,806,606 (80.89%)	22,693,622 (48.55%)	15,112,984 (32.33%)
JR3	52,747,306	46,500,712 (88.16%)	3,984,540 (7.55%)	42,516,172 (80.60%)	25,406,270 (48.17%)	17,109,902 (32.44%)
JS1	47,192,552	41,398,496 (87.72%)	4,096,332 (8.68%)	37,302,164 (79.04%)	24,983,358 (52.94%)	12,318,806 (26.10%)
JS2	59,305,266	52,746,872 (88.94%)	4,585,296 (7.73%)	48,161,576 (81.21%)	30,527,518 (51.48%)	17,634,058 (29.73%)
JS3	39,541,244	34,511,674 (87.28%)	1,612,788 (4.08%)	32,898,886 (83.20%)	21,429,523 (54.20%)	11,469,363 (29.01%)

JR1, JR2, and JR3 represent three independent biological replicates of the CSR-resistant cultivar Qi319; JS1, JS2, and JS3 represent three independent biological replicates of the CSR-susceptible cultivar Ye478.

## Data Availability

Sequencing data in this study can be found in the open repository Zenodo (https://doi.org/10.5281/zenodo.17938657, Published 15 December 2025).

## Supplementary Materials

The following are available online at https://www.mdpi.com/article/10.3390/biology15212033/s1, Table S1: Primers information of the genes for qRT-PCR. Table S2: Statistical table of the number of genes in different expression level intervals.
