# Supplementary material for: Comparative Transcriptome Analysis Identifies Candidate Coordinated Defense Mechanisms in Corn Stalk Rot Resistance
Source: Biology (Basel). 2026 Nov 8;15(21):2033. doi: 10.3390/biology15212033 (PMC13761602; doi:10.3390/biology15212033)
Supplement: Supplementary file 1 [file biology-15-02033-s001.zip › biology-4662083-supplementary.pdf]

Table S1. Primers information of the genes for qRT-PCR.

Primers	5'to3'	3'to5'
Zm00001d006069	AAGGTGCGTGCGTTGATAC	CGAGCGGAGAAAGCAATGTT
Zm00001d044122	CTCTGATTCGCTCCGTGTA	CATCAATGGCTTCGTCTTCC
Zm00001d001960	TGTTGTCAGTGTCAGTGC	CTGCTTCTGCTGCTGTAT
Zm00001d014914	GCTCAAGATCAACTACTACC	TTGTGGAGGATGAAGGAG
Zm00001d019850	CCAGCATCATCACAAGCAT	GCGTAGCCAATGAGGATG
Zm00001d027837	CCTTGCCATTGCCAACAT	GTCCAGCGTCATCCTCAT
Zm00001d014055	TCGGCAACTTCAAGGTAG	GTGAGCAGAAGACGACTT
Zm00001d051932	GGTGGATGAACGAAGAAGAAGAAG	GCAGGGCAGGCAACAAAT

Table S2. Statistical table of the number of genes in different expression level intervals.

Sample	0<=FPKM<1	1<=FPKM<5	5<=FPKM<10	10<=FPKM<30	30<=FPKM<50	50<=FPKM
JR1	15162(45.72%)	7738(23.33%)	3738(11.27%)	3989(12.03%)	969(2.92%)	1566(4.72%)
JR2	15427(46.52%)	7873(23.74%)	3613(10.9%)	3632(10.95%)	961(2.9%)	1656(4.99%)
JR3	16607(50.08%)	7524(22.69%)	3344(10.08%)	3510(10.58%)	803(2.42%)	1374(4.14%)
JS1	13853(41.77%)	6989(21.08%)	4108(12.39%)	5124(15.45%)	1241(3.74%)	1847(5.57%)
JS2	15221(45.9%)	7765(23.42%)	3702(11.16%)	3836(11.57%)	947(2.86%)	1691(5.1%)
JS3	14806(44.65%)	6551(19.75%)	3943(11.89%)	4750(14.32%)	1321(3.98%)	1791(5.4%)
